# Dopamine Use in Intensive Care: Are We Ready to Turn it Down?

**Published:** 2012-10-11

**Authors:** Geremia Zito Marinosci, Edoardo De Robertis, Giuseppe De Benedictis, Ornella Piazza

**Affiliations:** ^ Dipartimento di Scienze Chirurgiche Anestesiologiche e dell’Emergenza “G. Zannini”, Università degli Studi di Napoli “Federico II”, Naples, Italy; * Dipartimento di Medicina e Chirurgia, Università degli Studi di Salerno, Salerno, Italy

**Keywords:** dopamine, ICU, vasopressor, shock

## Abstract

Dopamine is still frequently used as a first line vasopressor agent in hypotensive patients, when physicians are afraid of noradrenaline and believe that dopamine, with its β and α, inotrope and vasopressor effects, may be helpful. Evidence exists that it does not offer protection from renal failure, even if at low doses (0, 3–5 mcg/Kg/min) it may exert its effects on D1 and D2 receptors resulting in natriuresis and renal vasodilation, augmentation in renal blood flow, and diuresis.

The effects of dopamine on gastrointestinal system and splanchnic perfusion in critical care patients are even more controversial, since they seem to be at least partially dependent on the initial fractional splanchnic blood flow. Dopamine may exert deleterious effects on respiratory function, by impairing the ventilatory drive response to hypoxemia and hypercapnia and reducing arterial oxygen saturation through a regional ventilation/perfusion mismatching. Dopamine seems to affect the cellular mediated mechanism of the immune function directly by its action on receptors located on immune system cells and indirectly altering the hormonal response regulating immune response. In this paper, the use of low dose dopamine is discussed in the intensive care perspective.

## I. INTRODUCTION

Dopamine (DA) remains an essential drug in Intensive Care Units (ICU), where it is still used as a first line vasopressor agent in hypotensive patients, refractory to fluid resuscitation, because of the feared ischemic side-effects of norepinephrine on end-organ perfusion.

Nevertheless, the results of the SOAP study showed that dopamine administration in shock patients, compared to patients who did not receive it, was associated with 20 % increase in ICU and hospital mortality rates [1]. Alternatives to dopamine infusion exist, as noradrenaline i.e. or other vasopressors, and informed guideline for sepsis management are available worldwide even if with some concerns are raised [2, 3].

Pharmacology of dopamine is interesting: its peculiar spectrum of action resides in the dose dependent interaction to different catecholamine receptors.

At low doses (0.3–5 μg/Kg/min) DA exerts its effects on D1 and D2 receptors resulting in, as the kidney is concerned, natriuresis and renal vasodilation, augmentation in renal blood flow, and diuresis.

Dose dependent interactions on different receptors are not a clear cut-off value but represent the prevalence of activation of a group of receptors over another with a wide range of inter-individual variability. Thus, even a low dosage of dopamine may exert a systemic vasocostrictory action without relevant improvement in renal function [4]. A low dosage of dopamine may even jeopardize mucosal blood flow in the gut, suppress the function of the pituitary gland, interfere with cell mediated immunity and impair the thyroid function [5–7].

The optimal selection of dopamine dosages is far less clear in critical care settings where an altered receptor function and responsiveness make the individual response unpredictable.

## II. RENAL EFFECTS

The era of dopamine, particularly “low dose dopamine” (LDD), began in 60’s when Goldberg described its effects on four patients affected by end stage congestive heart failure [8]. Drug administration, in doses ranging from 100 to 1,000 mcg/min, increased cardiac output and sodium urinary excretion. This phenomenon occurred at lower doses, and with minimal impact on cardiovascular status.

The same investigators showed that dopamine administration was able to increase plasmatic flow in the kidney, glomerular filtration, and sodium excretion in healthy human subjects [9]. In this study, the dose administered was titrated to achieve maximal renal effect without increasing mean arterial pressure.

In 1965, the same authors investigated the renal effects of dopamine in anaesthetised dogs and concluded that dopamine might exert its action on particular receptors located in the kidneys [10]. Twenty years after research by D’Orio et al., a series of dose response curves, based on renal and haemodynamic effects observed in patients to whom different doses of dopamine were administered, were observed [11].

The dopamine suppressor dose was at that time defined as the dose at which dopaminergic and possibly adrenergic stimulation prevailed over adrenergic stimulation. This threshold corresponded to the infusion rate: < 5g/kg/min [11].

Dopamine exerts its effects on the kidneys in dose dependent fashion.

At low doses, such as 0.3–5 μg/Kg/min, dopamine acts on D1 vascular receptors, which in turn increases renal blood flow. It appears that dopamine may additionally interact with D2 receptors located on presynaptic nerve endings, inhibiting the release of norepinephrine [12]. At higher doses, when adrenergic stimulation prevails, renal blood flow is augmented by the increase in cardiac output.

Dopamine is able to induce diuresis and natriuresis by acting on both D1 and D2 receptors located on the proximal tubule, which is the thick ascending loop of the Henle and cortical collecting tubule.

Those effects are achieved by the inhibition of Na^+^/K^+^-adenosine triphosphatase activity. In fact, it appears that the primary effect on renal epithelial cells is the removal of the plasma membrane of active Na^+^/K^+^ ATPase units. The net effect is the reduced capability of the tubular cells to Na^+^ transport [13].

Moreover, the stimulation of D2 receptors located on the collecting tubules of the inner medulla stimulates production of prostaglandin E_2_, (PGE_2_), which counterbalances the effects of antidiuretic hormones, augmenting the clearance of free water [14, 15].

The renal vasodilatory effects are associated with dose-dependent augmentation in renal blood flow and diuresis.

LDD induces a redistribution of intraparenchymal renal blood flow towards the cortical region, counteracting the effect of PGE_2_ and shunting blood away from the outer medulla [16].

This can be harmful for two reasons. First, renal medulla has a limited blood supply. Second, it may promote a relative ischemia in a region that is high metabolically active and already works with a lower tension of oxygen.

In fact, although the kidneys receive nearly 20 per cent of cardiac output, the greatest part of the blood flow supplies the outer parenchymal layers [17].

For years LDD was a widely accepted therapeutic option to limit or prevent acute renal failure in critical care patients, especially those affected by sepsis. Even if largely studied, sepsis is still the greatest danger for these patients’ life, with many obscure sides on its presentation, causes and prevention possibilities [18, 19]. Several investigations were carried out to assess the effects of LDD on renal function in critical patients who were at risk or had established renal failure.

In some studies, LDD administration increased urine output; however, in others, no effect was found [11, 20–23].

One study showed a potential negative effect of LDD dopamine administration on tubular function caused by the augmented urinary excretion of retinol binding protein in patients who had undergone coronary bypass surgery [22]. Another paper showed that in post-cardiac surgery, patients with normal preoperative renal function, dopamine was reported to increase renal oxygenation without increasing glomerular filtration rate, tubular sodium reabsorption, or renal oxygen consumption [24].

In fact, there is convincing evidence from literature that LDD not only is unable to prevent, reverse, or limit the progression of acute renal failure (ARF), but its use, regardless of a clear assessment of the volemic status of the patients, may increase the risk of ARF.

Moreover, a large prospective randomized study by the Australian and New Zealand Intensive Care Society Group showed that LDD not only was unable to prevent or reverse acute renal failure, but it failed to improve outcome variables.

In fact, there were no differences in terms of mortality, need of renal replacement therapy, renal recovery, and peak serum creatinine among the patients.

These findings confirmed the results of the retrospective analysis of the North American Septic Shock Trial (NORASEPT), where no reduction of the incidence of acute renal failure, the 28-day mortality, nor the requirement of haemodialysis were observed in septic patients who developed oliguria [25].

In two recent meta-analyses about the impacts of LDD on ARF, the first by Kellum and Decker, dopamine did not prevent mortality, the onset of acute renal failure, or the need for haemodialysis [26]. The second, by Marik, analysed 15 randomised controlled studies by comparing LDD administration with a placebo; there were no beneficial results in terms of serum creatinine change and incidence of acute renal failure [27].

It has been argued by some authors that adding LDD in patients requiring norepinephrine may limit its adverse effects on renal circulation and function.

Clear beneficial evidence on renal function of this therapeutic regime is lacking, as shown by studies carried out on experimental animal models and in patients with septic shock who require catecholamine administration.

It seems clear that LDD mediated increases in urinary output in septic shock patients treated with norepinephrine are probably mediated by the augmentation of cardiac output.

Recent evidence has shown that norepinephrine administration can effectively restore an adequate hemodynamic status in adequately fluid resuscitated patients [28].

The use of norepinephrine has been shown to have a protective effect on renal blood flow and to increase diuresis in animal and human septic shock conditions.

A low dosage dopamine appears to be able to increase in urinary output in critically ill patients, but it doesn’t play any protective role against acute renal failure and does not improve the course of an established acute renal failure.

When administered to critical patients, it may increase the risk of acute renal failure.

It could be interesting, but far from the topic of this review, to consider the use of new molecules in combination with dopamine, as vaptans i.e [29].

## III. GUT AND MESENTERIC EFFECTS

Gut has been considered as the “motor” of systemic inflammatory response syndrome (SIRS) [30]. In fact, alterations of mesenteric blood flow and gut hypoperfusion represent the first response to hemodynamic derangements in critically ill patients when blood, pooled away from intestinal viscera, is redistributed to “vital” organs. This response causes intestinal hypoperfusion that facilitates the alteration of the barrier function and the increase of intestinal epithelial apopotosis [31].

In experimental models, dopamine increased both splanchnic ad hepatic blood flow. In a study on dogs, dopamine reduced intestinal blood flow, and in a porcine model, it seemed to hasten gut ischemia.

Those results seem to be due to the ability of DA to reduce blood flow to the mucosa by redistributing it within the gut.

In another animal study DA improved mucosal blood flow and oxygenation [32].

Data regarding human studies shared the same deal of equivocal conclusion.

In fact, some investigations showed that LDD can increase splanchnic blood flow in septic cardiac surgical patients, whereas others did not draw the same results [33–35].

LDD seemed to decrease splanchnic oxygen consumption in septic patients in spite of an increase in splanchnic blood flow, and once again this effect was not confirmed in cardiac surgical patients.

LDD increased oxygen transport in septic patients but led to a diminished gastric mucosal flow and did not affect pHi, a common and widely accepted marker of gut mucosal perfusion.

The effect of DA administration seems to be at least partially dependent on the initial fractional splanchnic blood.

Recently, De Backer *et al*. found no differences in PCO2 gap, splanchnic blood flow in their study, which was carried out on 20 septic patients. Moreover, dopamine administration showed a lower mixed venous-hepatic venous saturation gradient.

DA 2 receptors are present in human enteric nervous endings, and dopamine administration may actually affect gastrointestinal motility.

These effects have been confirmed both in healthy subjects who had undergone short-term DA administration, and in critically ill patients, in doses ranging from 2.5 to 5 μg/kg/min. Moreover, in another paper, LDD impaired gastroduodenal emptying in mechanically ventilated patient during fasting and nasogastric enteral feeding [36]. Moreover there are data suggesting that norepinephrine does not impair splanchnic circulation in animal models of endotoxin shock and in septic patients. On the contrary, norepinephrine was associated with a greater increase in pHi as compared to dopamine in septic shock patients. Again, dopamine (4μg/kg/min) reduced hepatosplanchnic oxygen uptake in spite of an increase in systemic and regional perfusion. This effect was not shared by dobutamine.

## IV. RESPIRATORY EFFECTS

Intensive care patients are very often mechanically ventilated being respiratory failure the cause of their recovery in ICU or a complication of their illness [37–40]. As pointed out, DA administration may exert deleterious effects on respiratory functions. It impairs the ventilatory drive response to hypoxemia and hypercapnia by depressing the carotid body. It further reduces arterial oxygen saturation through a regional ventilation/perfusion mismatching. This does not represent a problem as long as patients are mechanically ventilated and an oxygen supplement is administered.

Problems can arise during the weaning process from ventilatory support, when the physiological response to both hypoxia and hypercapnia might have been blunted by DA administration. LDD may favour weaning from the ventilator, but this comes at the expense of an actual risk of hastening respiratory failure.

## V. ENDOCRINE AND IMMUNOLOGICAL EFFECTS

Low doses of dopamine result in plasmatic levels up to 100 times higher than those generated by endogenous secretions, which may cause partial hypopituitarism in adults, infants, and children. In a work by Van den Berghe [41] on 12 polytrauma patients, LDD dopamine administration lowered levels of thyroid stimulating hormones, thyroxine, and triiodothyronine. All values came back to normal after 24 hours from the suspension of DA.

LDD infusion may trigger or exacerbate the euthyroid sick syndrome in critical illnesses.

DA administration affected the secretion of the growth hormone (GH) in critically ill patients as pointed out by the same authors. GH pulsatile secretion is impaired in critically ill patients and it resulted further by being blunted by DA administration. The authors concluded that the suppression of GH secretion might enhance the catabolic process of critical illnesses.

LDD dopamine has been show to suppress dehydroepiandrosterone (DHEAS) and prolactine levels in 20 critically ill patients [41].

Cortisol levels were not affected. The levels of luteinizing hormone (LH) and testosterone were affected by dopamine administration in 15 critically ill men.

LH rose after three hours from dopamine withdrawal, while testosterone levels failed to rebound.

Dopamine receptors have been discovered on thymocytes, and dopamine is able to interact with lymphocytes. Dopaminergic agonist and dopamine suppress T-lymphocytes function and suppress T-cell functions in mice. In humans, specifically critically ill patients receiving dopamine, the drug was able to reduce the T-cells’ responsiveness

Prolactine, whose levels have been shown to decrease under dopamine infusion, has immunoregolatory functions; B and T types have indeed prolactine receptors.

The reduction of DHEAS has been advocated as a further cause of immune cellular response, because of lymphocyte T-helper and type 1-T lymphocyte induced dysfunction.

Dopamine seems to affect the cellular mediated mechanism of the immune function directly by its action on receptors located on immune system cells and indirectly altering the hormonal response regulating immune response.

## VI. CONCLUSION

Dopamine “owes” its popularity to the work of Goldberg. Over the years, renal and splanchnic protective effects have been challenged and not confirmed at all. There is no equivocal conclusion about its effects on gut, but DA has been proven to cause major disturbances in anterior pituitary function and the immune system and may further impair muscular blood supply.

We can conclude that low dose dopamine for renal protection is no longer justified and should be definitely abandoned. Its use as a first choice vasopressor should be questioned in view of its potentially deleterious side effects and the increased rate of mortality associated with its administration in septic patients. However, DA is still used by medical personnel; many doctors are familiar with the medication and feel comfortable for its application, therefore, it becomes part of their routine treatment: in clinical situations, very often, the choice of catecholamine is based on personal and cultural preferences, not evidence based.
